# Cervical Cancer Screening in the HPV-Vaccinated and Digital Era: Reassessing Strategies in Light of Artificial Intelligence and Evolving Risk

**DOI:** 10.3390/cancers17193179

**Published:** 2025-09-30

**Authors:** Apostolia Galani, Athanasios Zikopoulos, Efthalia Moustakli, Anastasios Potiris, Maria Paraskevaidi, Ioannis Arkoulis, Pavlos Machairoudias, Stefania Maneta Stavrakaki, Maria Kyrgiou, Sofoklis Stavros

**Affiliations:** 1 Department of Metabolism, Digestion and Reproduction, Faculty of Medicine, Imperial College London, London W12 ONN, UK; l.galani22@imperial.ac.uk (A.G.); m.paraskevaidi@imperial.ac.uk (M.P.); s.maneta-stavrakaki17@imperial.ac.uk (S.M.S.); m.kyrgiou@imperial.ac.uk (M.K.); 2 Third Department of Obstetrics and Gynecology, University General Hospital “ATTIKON”, Medical School, National and Kapodistrian University of Athens, 12462 Athens, Greece; thanzik92@gmail.com (A.Z.); garkoylis@hotmail.com (I.A.); pavlosmach@gmail.com (P.M.); sfstavrou@med.uoa.gr (S.S.); 3 Department of Nursing, School of Health Sciences, University of Ioannina, 4th Kilometer National Highway Str. Ioannina-Athens, 45500 Ioannina, Greece; ef.moustakli@uoi.gr

**Keywords:** HPV vaccination, cervical cancer, digital health, telemedicine, self-sampling, risk-based screening, health equity

## Abstract

**Simple Summary:**

Cervical cancer is largely preventable, yet it remains a major health problem, especially in low- and middle-income countries. The number of precancerous lesions has significantly decreased due to the human papillomavirus (HPV) vaccine, which is altering the standards for screening. HPV-based testing with longer intervals and more individualized methods is replacing Pap smears in several programs. Simultaneously, emerging digital tools such as self-sampling, telemedicine, and artificial intelligence can improve the accuracy and accessibility of screening for marginalized women. However, these advances also have drawbacks, including concerns about data privacy, equity, and ensuring health systems are ready to adopt them effectively. This review highlights the potential and difficulties for modifying cervical cancer screening in the ensuing decades by summarizing the most recent data on HPV vaccination and digital technology.

**Abstract:**

**Background**: The widespread use of the human papillomavirus (HPV) vaccine and the rise in digital technologies like artificial intelligence (AI) are causing significant changes in the paradigms surrounding cervical cancer screening. **Objective**: This review addresses potential future paths toward risk-based, customized screening and prevention while summarizing the available data on how vaccination and digital innovation are changing cervical cancer screening methods. **Results**: A shift from cytology-based screening to HPV-based primary testing with longer intervals has been supported by the notable decrease in high-grade cervical lesions brought about by HPV vaccination. However, AI and digital health tools, such as digital colposcopy, automated cytology, telemedicine, and self-sampling, have the potential to increase diagnostic access, accuracy, and efficiency, especially in low-resource environments. Implementation hurdles, data security, and algorithmic bias are major obstacles. **Conclusions**: Digital platforms, molecular diagnostics, and vaccination histories must all be incorporated into screening methods in order to keep up with the decline in HPV-related illness. The elimination of cervical cancer could be accelerated and equality and efficiency increased with customized, risk-based algorithms.

## 1. Introduction

Cervical cancer remains a major global health issue, with an estimated 660,000 new cases and 350,000 deaths in 2022, the majority of which occurred in low- and middle-income countries (LMICs) [1]. These figures highlight the inequity in access to effective prevention services despite the disease being largely preventable. In high-income countries, organized cytology-based screening programs have significantly reduced cervical cancer incidence and mortality [2]. However, these traditional paradigms are being reshaped by two transformative developments: the global implementation of prophylactic HPV vaccination and the rise in digital health technologies, including artificial intelligence (AI) [3].

International health authorities have set high targets for cervical cancer elimination. By 2030, 90% of girls should be fully vaccinated against HPV by the age of 15, 70% of women should receive a high-performance test by the ages of 35 and 45, and 90% of women with precancerous lesions or invasive cervical cancer should receive appropriate treatment, according to the World Health Organization’s (WHO) 90-70-90 strategy. Scaling up established interventions and adapting them to the changing epidemiology influenced by vaccination are essential to meet these goals [1].

HPV vaccination, particularly when administered prior to sexual debut, is highly effective in reducing HPV 16 and 18 prevalence, which cause approximately 70% of cervical cancers [4,5,6]. Population-level data from countries with high vaccine uptake confirm dramatic declines in high-grade cervical lesions among vaccinated cohorts [7]. This changing epidemiology alters the positive predictive value of cytology, increasing the risk of overdiagnosis and unnecessary intervention [8,9,10]. Consequently, many countries have transitioned to primary HPV testing with longer screening intervals and revised age thresholds [11,12,13].

Cytology-based programs have historically been the mainstay of cervical cancer control since the invention of the Papanicolaou (Pap) smear in the middle of the 20th century [14]. Developments such as liquid-based cytology enhanced sample quality and enabled molecular HPV detection. However, declining frequencies of high-grade lesions challenge cytology effectiveness, making risk-based stratification and primary HPV testing increasingly attractive [15].

Digital innovation is transforming cervical cancer screening infrastructure and delivery. AI-powered algorithms can enhance diagnostic accuracy in cytology and colposcopy, while mobile health (mHealth) platforms, telemedicine, and self-sampling technologies can increase accessibility in under-screened populations [16,17,18,19,20]. These advances raise ethical and logistical concerns around algorithmic bias, data privacy, and health system readiness [21,22,23]. However, these benefits are contingent upon cultural acceptability, sufficient internet and technical infrastructure, algorithm validation across diverse populations, and trustworthy data systems. To ensure safe implementation, human oversight is still essential [24].

Early clinical studies suggest that AI-supported colposcopy and automated cytology platforms may reduce inter-observer variability and match or exceed human sensitivity [25]. Self-sampling combined with digital registries, cloud-supported telemedicine, and smartphone imaging are being tested to address workforce and infrastructure gaps in LMICs. When appropriately deployed, these technologies could reduce inequities in preventative access [26].

This review synthesizes evidence on HPV vaccination and digital innovation in cervical screening, highlighting the shift toward risk-based, personalized strategies. To educate effective and equitable cervical cancer prevention in the ensuing decades, it assesses the potential of AI and digital health tools while addressing implementation, ethical, and legal difficulties.

## 2. Methodology

This article is a narrative review that summarizes the most recent data on cervical cancer screening, HPV vaccination, and digital advancements. The following keyword combinations were used to search PubMed, Scopus, and Web of Science for English-language articles published between January 2010 and March 2025: “HPV vaccination,” “cervical cancer screening,” “primary HPV testing,” “self-sampling,” “digital health,” “telemedicine,” and “artificial intelligence.” Large observational studies, modeling studies, randomized controlled trials, systematic reviews, and significant policy publications (e.g., WHO guidelines) were prioritized. By tracking the citations of important articles, more references were found. The review is not a conventional systematic review, but it attempts to present a thorough, fair, and current summary.

## 3. The Impact of HPV Vaccination on Cervical Cancer Risk

Cervical cancer prevention now relies heavily on HPV vaccination, screening, and early treatment. The influence of vaccination varies globally due to differences in introduction, rollout, and coverage, and it interacts with screening programs and epidemiological trends. Reductions in HPV prevalence, herd effects, screening implications, global disparities, and programmatic modifications are some of the important aspects of this impact that are described in the following subsections [27].

### 3.1. Reductions in HPV Prevalence and CIN

The introduction of HPV vaccines has significantly altered the epidemiology of cervical cancer. The bivalent, quadrivalent, and nonavalent vaccines have demonstrated high efficacy in preventing persistent infection with HPV types 16 and 18, and in the case of the nonavalent vaccine, five additional high-risk genotypes [28,29,30]. As a result, substantial reductions in vaccine-type HPV prevalence and high-grade cervical intraepithelial neoplasia (CIN2+) have been reported in countries with high vaccination coverage [31,32,33]. In Australia, the incidence of CIN2+ in women under 25 declined by over 50% within a decade of vaccination rollout [34]. In Scotland, vaccination at age 12–13 was associated with a near 90% reduction in CIN3+ [35].

According to long-term follow-up studies, there is no sign of declining immunity, and these protective effects last for at least 12 to 15 years. Furthermore, meta-analyses have verified notable decreases in anogenital warts, HPV prevalence, and cervical lesions in all vaccinated cohorts. These results demonstrate the widespread advantages of vaccination at the population level, especially when high coverage is attained in the early stages of adolescence.

### 3.2. Herd Immunity and Male Vaccination

The benefits of vaccination extend beyond direct protection. Evidence of herd immunity has emerged in populations where coverage is high, with declines in HPV prevalence and genital warts observed among unvaccinated individuals [36,37,38]. In settings that have adopted gender-neutral vaccination policies, such as Australia and the US, these effects have included reductions in HPV-related disease among heterosexual men and men who have sex with men (MSM), who are at heightened risk for anal, penile, and oropharyngeal cancers [39,40,41].

Whereas indirect protection is limited in areas with lower uptake, the effects of herd immunity are greatest in areas where female immunization rates exceed 70%. It has also been demonstrated that extending coverage to boys increases cost-effectiveness, especially when it comes to lowering HPV transmission and safeguarding high-risk populations. Notably, in many settings, vaccination of boys is still optional, which may restrict the effects on herds. Gender-neutral programs are becoming more and more supported by cost-effectiveness evaluations, especially when it comes to protecting MSM populations and lowering transmission [42]. This more comprehensive approach increases population-level resistance to several malignancies linked to HPV.

### 3.3. Screening Performance and Predictive Value

In vaccinated populations, the reduced prevalence of HPV-associated disease lowers the positive predictive value of cytology-based screening [43,44,45]. This can lead to more false positives and overtreatment, particularly if screening protocols are not adjusted. To address this, primary HPV testing has been adopted in many countries, often with extended intervals and age thresholds, supported by evidence from large modelling studies and population trials [46,47,48]. These new protocols often incorporate reflex triage strategies, such as partial genotyping and molecular biomarkers, to maintain specificity and minimize harm [49,50,51].

Instead of being a primary test in this context, cytology is increasingly being considered a secondary or triage technique. To increase specificity and risk classification, new biomarkers like p16/Ki-67 dual staining and DNA methylation are being investigated. In communities where the prevalence of disease is reducing, these improvements are essential for striking a balance between sensitivity and the need to prevent overtreatment.

### 3.4. Global Inequities in Vaccine Uptake

Despite these advances, global disparities in vaccine coverage remain stark. As of 2023, many LMICs have yet to introduce national HPV vaccination programs, and among those that have, coverage is often below target levels due to cost, supply chain issues, and vaccine hesitancy [52,53,54]. Current estimates of coverage are updated with recent data from 2024, which also shows enduring disparities. Disparities already present were exacerbated by the COVID-19 pandemic, which interfered with immunization schedules and screening services, especially in LMICs [55]. This creates a dual challenge for screening systems, which must accommodate both vaccinated and unvaccinated cohorts—often within the same population [21,56,57]. Flexible, risk-adapted screening strategies are therefore essential.

Inequities in the burden of cervical cancer are perpetuated by the fact that coverage is below 20% in many LMICs while exceeding 80% in several high-income nations. For instance, vaccination rates are over 80% in nations like Australia and the UK, but less than 20% in several LMICs, including certain countries in sub-Saharan Africa [58].

Platforms for school-based delivery have been successful in increasing access in some areas, but they risk leaving out-of-school teenagers behind. Though implementation issues still exist, recent WHO recommendations in favor of a single-dose regimen may aid in increasing global usage [59].

### 3.5. Implications for Screening Programs

The evolving impact of HPV vaccination necessitates rethinking screening approaches. In many high-income settings, screening now begins at age 25 or 30, and is conducted at 5–10-year intervals depending on individual risk [60,61,62]. Vaccination history, HPV genotype, and prior test results are increasingly used to guide screening intervals and triage decisions [63]. This shift from uniform to personalized screening is essential to balance benefit and harm as HPV-related disease becomes less common in vaccinated populations [64,65,66].

Currently, hybrid systems are needed, particularly in transitional contexts, to handle overlapping cohorts of people who have received vaccinations and those who have not. Tracking immunization status and incorporating it into screening algorithms is becoming more and more dependent on registries and digital health platforms. The future of cervical cancer prevention may ultimately be determined by risk-adapted models that are affected by both molecular diagnoses and immunization history [67].

## 4. Shifts in Screening Guidelines and Risk Stratification

The evolution of cervical cancer prevention strategies in the context of widespread HPV vaccination has prompted a fundamental shift in screening paradigms. Several high-income countries, including Australia, the UK, and the Netherlands, have transitioned from cytology-based screening to primary HPV testing, reflecting the increased sensitivity and predictive value of HPV detection in vaccinated populations [57,68].

National, centrally coordinated programs have frequently been used to accomplish these reforms, enabling methodical outcome tracking and protocol adaptation over time. As the incidence of HPV-related disease continues to decrease in successive vaccinated cohorts, preliminary data from these nations indicate that primary HPV testing is not only more sensitive but also more economical [69].

HPV testing detects persistent infection earlier than cytology can detect morphological abnormalities, allowing earlier intervention and reducing the risk of progression to high-grade disease or invasive cancer [70,71]. As the prevalence of high-risk HPV types declines among vaccinated cohorts, the relative benefits of cytology diminish, while the harms of overdiagnosis and unnecessary colposcopy increase [72]. These changes have spurred the development of more nuanced, risk-based approaches to screening that account for vaccination status, HPV genotype, age, prior screening history, and comorbid risk factors [73,74]. Crucially, this change highlights the importance of dynamic risk assessment rather than static screening intervals. Future screening programs may develop toward true precision-based models of prevention by integrating longitudinal data and individual risk factors, which would minimize needless treatments and protect against malignancies that are overlooked [75].

The move toward personalized screening has been supported by modelling studies demonstrating the cost-effectiveness and safety of extended screening intervals and delayed initiation of screening in vaccinated individuals. For example, evidence suggests that screening could safely begin at age 30 and be performed every 10 years in women who received the HPV vaccine before sexual debut [76,77]. In some jurisdictions, algorithms now integrate HPV genotyping (especially for types 16 and 18) and/or molecular triage markers to determine the need for immediate colposcopy versus routine surveillance [78]. These tactics represent a significant paradigm change away from a “one-size-fits-all” approach and toward stratified prevention, in which the level of follow-up is tailored to each individual’s risk. Though promising, they need top-notch laboratory facilities and registries to guarantee precise application and reduce the possibility of underscreening in susceptible populations [79].

In addition to algorithmic changes, guidelines have also evolved regarding the method of sample collection. Self-sampling for HPV testing is increasingly recognized as an effective and acceptable alternative to clinician-collected samples, particularly for underscreened populations [80]. This approach can enhance accessibility and equity, especially in rural, underserved, or culturally marginalized communities [81]. Implementation studies and randomized trials verify that, when using high-quality assays, self-sampling yields sensitivity comparable to that of clinician-collected samples. Furthermore, in LMICs, where screening participation is frequently hampered by cultural barriers and a lack of clinical capability, incorporating self-sampling into national programs may prove revolutionary [82]. Self-sampling increases participation, but challenges remain, such as managing follow-up of positive results, ensuring sample adequacy, and addressing patient concerns about performing properly. Consideration must also be given to logistics for shipping kits and guaranteeing lab capacity [83].

Despite these advances, there remains considerable heterogeneity in global screening practices. In many LMICs, cytology remains the primary method, and screening coverage is low due to limited infrastructure, workforce shortages, and sociocultural barriers [68,84]. To bridge this gap, international organizations advocate for the integration of scalable technologies, simplified protocols, and task-shifting models to optimize delivery in resource-limited settings [85]. In comparison to HPV testing, visual examination with acetic acid (VIA) is still the standard in many LMICs, but its sensitivity and specificity are lower. The future will present a challenge in introducing cost-effective, scalable, and flexible HPV-based strategies while making sure that advancements in technology do not worsen global disparities in cervical cancer prevention [86].

Risk-based screening represents a paradigm shift toward precision prevention, offering the promise of more efficient and equitable care. However, its implementation requires robust data systems, interdisciplinary coordination, and public engagement to ensure safety, acceptance, and sustainability [87]. To prevent misunderstandings or decreased involvement, it will be essential to maintain community trust and communicate clearly about changing guidelines. Ultimately, political will, resource allocation, and a persistent dedication to equality will be just as important to the success of risk-stratified models as technological prowess [88].

**Figure 1:**
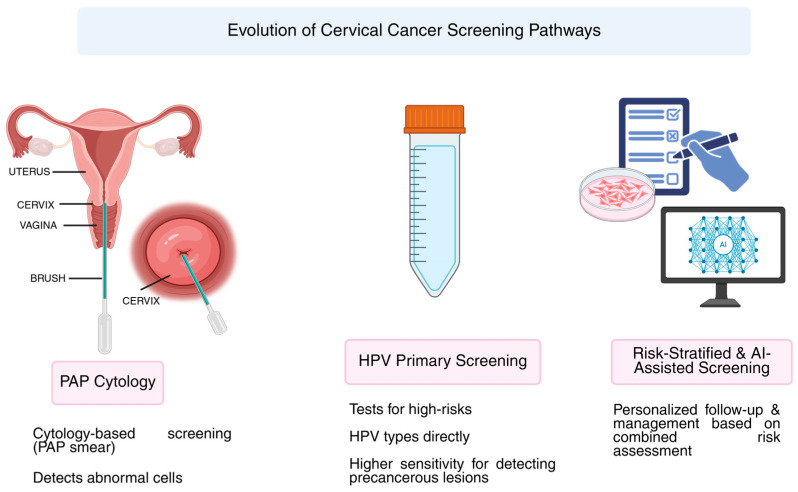
The figure illustrates the transition from cytology-based screening (Pap smear) toward primary HPV testing and, ultimately, risk-stratified and AI-assisted models.

Practical difficulties emerge when adding vaccination history into screening algorithms, especially in LMICs lacking centralized immunization databases. Risk misclassification may happen due to manual records or memory bias. While requiring infrastructure and political support, digital solutions like using unique patient IDs or linking EHRs with school immunization records look promising. Scalable models should draw on lessons from countries with strong registries, such as Denmark and Australia, for resource-limited settings [89]. Figure 1 summarizes the evolution of cervical cancer screening pathways from cytology-based programs toward primary HPV testing and risk-stratified, AI-assisted models.

## 5. Integration of Artificial Intelligence in Screening

AI is increasingly being explored as a transformative tool in cervical cancer screening, promising enhanced diagnostic accuracy, automation, and scalability, particularly in the context of declining disease prevalence and strained health systems. AI-based tools have demonstrated potential across the screening continuum—from cytology and colposcopy interpretation to workflow triage and patient follow-up [90,91].

AI is being considered as a way to increase efficiency in settings with limited resources, as well as a diagnostic supplement as health systems adjust to the changing burdens of disease. Incorporating AI-powered solutions could eventually lower the cost per screen, increase coverage, and standardize quality in an array of contexts [92].

### 5.1. AI in Cytology and Histology Interpretation

Conventional cytology is labor-intensive and subject to human error, especially in low-prevalence settings where abnormal findings are rare and fatigue can affect diagnostic accuracy [90,93]. Deep learning algorithms trained on large image datasets can now match or exceed expert-level performance in detecting cervical abnormalities in digitized Pap smears and liquid-based cytology (LBC) samples [94,95,96]. In one large retrospective analysis, an AI-enabled cytology platform demonstrated over 90% sensitivity for high-grade lesions, with significantly reduced false positives [97]. These developments may allow regional facilities to use AI-assisted platforms to make up for staff shortages, while central laboratories may be able to process massive sample volumes more consistently. Significantly, AI may also reduce intra- and inter-observer variability in cytological reporting, acting as a quality control tool [98].

AI can also be used to support histopathological diagnosis by standardizing grading of CIN lesions, which often suffer from high inter-observer variability [99]. Automated image analysis may enable more consistent and reproducible interpretation across settings, particularly where trained pathologists are scarce. By providing real-time feedback and annotated reference cases, AI-assisted histology could accelerate the training of early-career doctors. In regions with limited access to expert pathologists, particularly outside major urban centers, this approach may also strengthen diagnostic capacity in LMICs [100].

### 5.2. AI in Colposcopy and Visual Assessment

AI models using computer vision and convolutional neural networks (CNNs) have shown promising results in digital colposcopy. These systems can analyze cervical images in real-time to identify areas of concern, guide biopsy, and support clinical decisions [101,102]. Mobile colposcopy combined with AI may be particularly impactful in LMICs, where it can compensate for limited specialist availability [103]. The 90-70-90 cervical cancer elimination targets set by the WHO are closely aligned with such advancements. By utilizing widely accessible mobile technology, AI-enabled solutions could help community health workers deliver services beyond formal clinical settings [104].

Several platforms now integrate AI tools into smartphone-based or handheld colposcopy devices, providing portable, low-cost solutions for use in community and outreach programs [105].

### 5.3. AI for Risk Stratification and Screening Workflow Optimisation

Beyond image analysis, AI algorithms can support broader decision-making by integrating data from multiple sources—HPV genotype, cytology, patient history, and sociodemographic risk factors—to generate individualised risk scores [106]. These models can guide screening intervals, triage decisions, and resource allocation [107]. In health systems with limited capacity, AI tools may help prioritise high-risk patients and reduce unnecessary referrals, improving efficiency [108]. Under-treatment and overtreatment may be decreased by matching screening intensity to actual risk using this data-driven method. Long-term, risk profiles might be updated continuously as new clinical data becomes available by integrating predictive AI algorithms into electronic health records [109].

Some pilot programs have tested AI-assisted risk triage using natural language processing (NLP) and machine learning to process electronic health records and flag overdue or high-risk individuals [71]. While promising, such applications raise questions about data quality, interoperability, and digital infrastructure in diverse health systems. According to implementation studies, combining self-sampling with digital navigation platforms and community outreach can be a practical way to enhance equity in areas with the greatest cervical cancer burden; nevertheless, local adaptation and consistent funding are essential [110]. Adequate digital literacy among patients and healthcare professionals is another requirement for implementation. The safety of risk-based screening algorithms may be jeopardized if reliance on fragmented or partial datasets is not carefully planned for [111].

### 5.4. Limitations and Ethical Considerations

Despite their potential, AI tools face challenges related to algorithmic bias, generalizability, and regulatory oversight. Many models are trained on datasets that may not be representative of all populations, raising concerns about inequities in diagnostic performance [112,113,114]. AI systems must be validated across diverse ethnic, geographic, and socioeconomic settings to avoid reinforcing existing disparities in screening access and outcomes [115]. For instance, regional variations in comorbidity patterns, HPV subtype distribution, and cervical imaging quality may all have an impact on algorithm performance. Certain subpopulations run the danger of being systematically underdiagnosed in the absence of representative training data [116].

Furthermore, integration into clinical practice requires rigorous regulatory approval, ethical safeguards, data governance structures, and public trust. Concerns about data privacy, particularly in the context of reproductive health, are especially salient [117]. Human oversight remains essential to ensure AI is used as a decision-support tool rather than a replacement for clinical judgment [118]. To establish long-lasting trust, it will be essential to disclose algorithm performance transparently, make decision-making processes understandable, and involve patients in governance structures. Ultimately, AI in cervical screening should be viewed as an addition to, not a replacement for, knowledgeable clinical knowledge [119].

### 5.5. Discrepancies and Clinical Liability in AI Use

Clinical governance is called into doubt when AI outputs and expert interpretations diverge, even if AI technologies may be as accurate as humans or more so. Despite being typically low (less than 5%), studies have demonstrated that discordance rates might have an impact on clinical judgment and patient trust. In pilot implementations, it is advised to have a clear escalation channel, such as mandating human re-review in the event of discordance [120].

There is still some ambiguity about liability in AI misdiagnosis; developers, healthcare systems, and physicians may all have some of the blame. To reduce risk, emerging frameworks, such as the EU AI Act, place a strong emphasis on human-in-the-loop supervision and documentation of decision-making processes. AI-assisted misclassification case reports highlight the importance of real-world monitoring and prospective validation before widespread use (Table 1) [121].

## 6. Digital Health Tools and Screening Access

Digital health technologies—including mobile health (mHealth), telemedicine, electronic health records (EHRs), and digital decision-support platforms—are increasingly integral to the reorganization of cervical cancer screening, particularly in under-resourced settings. These tools offer scalable solutions to improve access, engagement, and continuity of care, especially for populations historically underserved by traditional healthcare infrastructure [122,123].

### 6.1. Mobile Health (mHealth) and Text-Based Reminders

Mobile-based interventions, such as SMS reminders, have been shown to increase screening uptake, reduce missed appointments, and improve follow-up adherence. In LMICs, widespread mobile phone access makes SMS strategies cost-effective and feasible at scale. Programs in Kenya, India, and Peru demonstrated notable gains in screening coverage and patient return rates using mHealth tools [124].

Mobile apps can also deliver culturally adapted education, promote HPV vaccination, guide self-sampling, and connect women with local screening services. Combining education with scheduling tools and reminders has, in some populations, doubled screening participation [125].

### 6.2. Telemedicine and Digital Colposcopy

Telemedicine enables remote interpretation of cytology and colposcopy images, bridging gaps in specialist availability. Digital colposcopy systems can capture high-resolution images that are stored and transmitted via secure platforms for review by expert colposcopists, facilitating decentralized screening models [126]. In low-resource areas, where trained professionals are scarce, nurse-led or community-based screening with telemedicine support has proven effective in maintaining screening quality [127].

In high-income settings, telecolposcopy is increasingly used for triage, second opinions, or quality control, especially in the context of abnormal self-collected HPV results. Combined with AI-based interpretation tools, telemedicine has the potential to reduce diagnostic variability and wait times [128,129].

### 6.3. Digital Registries and Integrated Health Records

Digital registries and integrated EHRs are vital for the coordination and evaluation of screening programs. They enable tracking of screening status, vaccination history, test results, and follow-up compliance. In countries like Sweden, Denmark, and Australia, centralized digital registries have supported risk-based screening strategies and allowed continuous monitoring of program effectiveness [130].

Automated prompts within EHRs can assist clinicians in identifying eligible women and ensuring that screening is conducted according to the most recent guidelines. Decision-support tools that integrate risk scores, previous results, and vaccination data have also been piloted to enhance clinical workflow [131].

### 6.4. Barriers to Digital Implementation

Despite the promise of digital health tools, several challenges remain. These include disparities in digital literacy, limited internet infrastructure, concerns about data security, and resistance to technological change among providers and patients alike. In some contexts, the use of mobile or web-based tools may inadvertently exacerbate inequalities if not accompanied by efforts to promote accessibility and inclusivity [132].

Data privacy and informed consent are also major considerations, particularly when handling reproductive health data in environments where digital surveillance may pose legal or social risks. Ensuring ethical and transparent use of data is paramount, especially when deploying AI-enhanced or cloud-based systems [133,134]. All of these innovations show great promise, but they nevertheless face challenges (Table 2), underscoring the need for regulations that ensure both equitable access and technological effectiveness. Research supports multilevel treatments that, for maximum effect, combine digital reminders with more conventional outreach techniques, including public education campaigns and community health worker programs.

## 7. Equity, Ethics, and Implementation Challenges

The evolution of cervical cancer screening brings substantial clinical and technological advances but also raises complex ethical, social, and logistical challenges. Ensuring innovation translates into equitable outcomes requires attention to structural determinants of health, inclusive policy design, and ethical frameworks for data use and service delivery [135,136].

Digital innovations may exacerbate existing disparities if they are scaled without clear equity frameworks. In LMIC contexts, for example, AI algorithms trained on high-income population data may perform poorly, while smartphone-based self-sampling programs may not include women without digital access [137]. Digital navigation combined with community outreach enhances sustainability and equity, according to real-world research from Kenya, India, and Peru. Future initiatives should incorporate equality criteria into implementation monitoring, such as low-income or rural groups’ engagement [138].

### 7.1. Unequal Access to Innovation

Disparities in screening persist both within and between countries. In high-income settings, women of low socioeconomic status, recent immigrants, indigenous populations, and those in remote areas are less likely to be screened. In LMICs, fewer than 20% of women undergo routine screening, primarily due to limited infrastructure, awareness, and trained personnel [136].

Technologies such as self-sampling and AI-assisted diagnostics could reduce access barriers, but only if implementation actively includes vulnerable populations. Without strategies to address linguistic, cultural, digital, and geographic divides, these tools may inadvertently widen existing gaps [139].

### 7.2. Ethical Implications of AI and Digital Tools

The integration of AI in screening raises important ethical concerns. Algorithms trained on datasets from predominantly white, urban populations may not generalize well to ethnically diverse or underserved groups. Algorithmic bias may lead to systematic under- or over-diagnosis if models are not externally validated across demographic strata [140]. 

Regulatory frameworks remain limited or fragmented in many countries, complicating safe deployment. Rigorous technical validation, including internal and external testing across populations and settings, is essential. Guidelines now emphasize transparency, reproducibility, and independent evaluation before clinical adoption [141].

### 7.3. Data Privacy and Informed Consent

Digital tools often involve sensitive health data, including sexual and reproductive health information. Ensuring informed consent is crucial, particularly in contexts with low literacy or limited digital familiarity [142]. Risks of data misuse are heightened in regions with weak privacy laws or punitive regulations. Adoption of privacy-preserving technologies and global standards for responsible data stewardship is essential as digital platforms scale [143].

### 7.4. Workforce and Health System Capacity

New screening technologies require a trained workforce capable of operation, maintenance, and interpretation. In resource-limited settings, task-shifting to nurses or community health workers can be effective but demands investment in training, supervision, and quality assurance. In high-income countries, resistance may arise due to workflow disruption, reimbursement concerns, or liability issues. Implementation science approaches are critical to ensure innovations are contextually adapted, sustainable, and integrated within existing health systems [85,144].

## 8. Future Directions and Research Gaps

As cervical cancer prevention enters a new era shaped by vaccination, risk stratification, and digital technologies, the research agenda must evolve to ensure policies and practices remain aligned with emerging needs. Key priorities include evaluating long-term vaccine effectiveness, refining screening algorithms, evaluating new technologies, and addressing persistent disparities in access and outcomes [135,145].

### 8.1. Long-Term Impact of HPV Vaccination

While early data are promising, longer-term surveillance is needed to assess the durability of vaccine-induced immunity, potential genotype replacement, and the eventual impact on cervical cancer incidence and mortality. Large population-based registries will be critical for tracking vaccinated cohorts into older age and informing adjustments to screening policies [146,147].

The efficacy of single-dose HPV vaccination—now endorsed by WHO for certain populations—also requires longitudinal evaluation, particularly in LMICs where it could substantially increase coverage [148]. Key research gaps and priorities are summarized in Table 3, outlining directions for vaccination surveillance, optimized screening, digital technologies, equity, and cost-effectiveness.

### 8.2. Optimizing Screening in Vaccinated Populations

As vaccine-protected cohorts reach screening age, reassessment of screening frequency, starting age, and triage protocols is urgent. Trials and modeling studies are informing these adjustments, but real-world evidence remains limited. Research should validate the safety and cost-effectiveness of extended screening intervals (e.g., 10 years) and clarify how to integrate vaccination history into clinical algorithms [149,150].

The role of HPV genotyping, molecular markers (e.g., methylation, E6/E7 mRNA), and multi-modal triage strategies warrants further study. These tools could enable refined risk stratification, particularly in populations with mixed vaccination status [151].

### 8.3. Validation of AI and Digital Tools

AI and digital platforms offer enormous potential, but few have undergone prospective clinical trials or external validation in diverse populations. Further research is needed to assess performance across different ethnicities, settings, and disease prevalence levels. Regulatory frameworks must evolve in parallel to ensure safety, transparency, and accountability [152,153,154].

Usability and implementation studies are essential to guide clinical integration, ensuring tools are acceptable to clinicians and patients and enhance, rather than disrupt, workflow [155].

### 8.4. Self-Sampling, Equity, and Outreach

Evidence supports self-sampling, but operational challenges remain for large-scale implementation, especially in LMICs and remote areas. Future research should explore models combining self-sampling with digital reminders, tele-triage, or community-based navigation [110].

Understanding how digital innovations influence health equity—positively and negatively—is also critical. Disaggregated data by socioeconomic status, geography, ethnicity, and other factors will help monitor equity impacts over time [156].

### 8.5. Cost-Effectiveness and Health Systems Integration

Comprehensive economic evaluations are needed to assess the cost-effectiveness of emerging screening strategies in real-world settings, accounting for health system constraints, workforce requirements, and long-term outcomes. Models should compare traditional cytology and HPV-based screening with AI-supported, risk-stratified, and self-sampling approaches across different resource contexts [135].

### 8.6. Current Controversies and Evidence Gaps

Several concerns still exist despite compelling evidence in favor of digital innovation and HPV-based screening. According to certain studies, self-sampling is not as well accepted as anticipated in some populations. In a similar vein, AI-assisted screening cost-effectiveness models depend on optimistic assumptions on algorithm performance and infrastructure preparedness [128]. The necessity for careful deployment and ongoing monitoring is highlighted by unfavorable results, such as over-referral from overly sensitive AI models and false reassurance from false negatives in self-sampling. In partially vaccinated cohorts, there are still disagreements on the best age and interval to begin screening, highlighting the significance of country-specific modeling prior to changing policy [157].

## 9. Conclusions

Cervical cancer prevention is evolving rapidly. HPV vaccination has reshaped disease patterns, prompting a shift toward primary HPV testing and risk-based screening strategies that consider vaccination status, age, and HPV genotype. Digital health tools and AI offer opportunities for more personalized, efficient, and equitable screening, including self-sampling for under-screened populations.

However, these advances bring ethical, logistical, and implementation challenges. Ensuring equity, safeguarding data, and maintaining robust oversight are essential. Screening programs must remain adaptable, evidence-informed, and responsive to local contexts. With coordinated, thoughtful implementation, innovation can translate into real progress toward cervical cancer elimination.

## Figures and Tables

**Table 1:** Overview of digital health tools for cervical cancer screening: applications, supporting evidence, adoption readiness, and key limitations. Abbreviations: LMIC = low- and middle-income countries; HIC = high-income countries; EHR = electronic health record.

Tool/Application	Primary Function	Evidence Strength	Readiness for Adoption	Key Limitations
AI-assisted Cytology	Automated detection of abnormal cells	Multiple large retrospective studies, with some prospective validation	Moderate–High (available in some labs)	Dataset bias, need for human oversight
AI-supported Colposcopy	Real-time image classification and biopsy guidance	Early feasibility and pilot studies	Low–Moderate (mostly research stage)	Requires high-quality imaging, training data generalizability
Risk-stratification Algorithms	Integrate HPV genotype + history to guide follow-up	Modelling studies, some pilot implementations	Moderate (implemented in select programs)	Need for EHR integration, data privacy concerns
Digital Registries & EHR	Tracking vaccination, results, follow-up	Extensive real-world evidence in high-income settings	High (standard in many countries)	Infrastructure and cost barriers in LMICs
Telemedicine & Digital Colposcopy	Remote interpretation, quality control	Demonstrated feasibility in multiple LMIC pilots	Moderate	Internet connectivity, secure transmission, training

**Table 2:** Applications, evidence, and barriers of digital health tools for cervical cancer screening. Abbreviations: LMIC = low- and middle-income countries; HIC = high-income countries; SMS = short message service; EHR = electronic health record.

Tool	Key Functions	Evidence	Relevance for LMICs	Limitations
mHealth (SMS, apps)	Appointment reminders Education HPV vaccination promotion, Self-sampling guidance	SMS reminders increased uptake, reduced loss to follow-up; apps doubled participation in some populations [99,105]	Widespread mobile access enables cost-effective scale-up (Kenya, India, Peru) [102,103]	Digital literacy gaps, Shared phone use Privacy concerns [115,116,117,118]
Telemedicine & Digital Colposcopy	Remote interpretation of cytology/colposcopy, Tiage, quality control	Maintains diagnostic quality with nurse-led/community screening; supports second opinions in HICs [106,109]	Compensates for scarcity of specialists; facilitates decentralized screening [107,108]	Connectivity issues Data security Dimited infrastructure [115,116,117,118,119,120]
Digital Registries & EHRs	Centralized tracking of vaccination Screening, DFllow-up; automated clinician prompts	Supported risk-based strategies in Sweden, Denmark, Australia [110,111,112]; improved guideline adherence [113]	Facilitates monitoring and continuity, though adoption is limited by infrastructure	Requires robust IT systems, Data protection frameworks [115,116,117,118,119,120]
Decision-Support Tools	Integration of risk scores Prior results, vaccination data	Pilots show enhanced workflow and decision-making [114]	May guide non-specialist providers in LMICs	Early-stage implementation; interoperability challenges [115,116,117,118,119,120]
Cross-cutting barriers	—	—	May exacerbate inequalities without inclusive design [118]	Digital literacy Internet access Provider resistance Privacy/legal risks [115,116,117,118,119,120]

**Table 3:** This table outlines key research domains relevant to cervical cancer elimination, including HPV vaccination, screening in vaccinated cohorts, AI and digital tools, self-sampling, and health system integration.

Research Area	Key Questions	Expected Impact
Long-term HPV vaccination impact	What is the durability of immunity? Is there genotype replacement? How effective is single-dose vaccination in LMICs?	Inform vaccine policy and optimize global coverage
Screening in vaccinated cohorts	What is the safe starting age and interval? How should vaccination history be integrated?	Ensure effective, cost-efficient, risk-adapted screening
AI and digital tools	How do AI models perform in diverse populations? What regulatory and ethical safeguards are needed?	Enhance diagnostic accuracy, standardization, and scalability
Self-sampling and equity	How can self-sampling be scaled with digital support? What are its effects on underserved populations?	Improve participation and reduce disparities
Cost-effectiveness and systems integration	How do new strategies compare to traditional methods across settings?	Guide resource allocation and sustainable implementation

## Data Availability

Data is unavailable due to privacy or ethical restrictions.
